# Learning curve in minimally invasive mitral valve surgery: a single-center experience

**DOI:** 10.1186/s13019-019-1038-0

**Published:** 2019-12-05

**Authors:** Anh Tuan Vo, Dinh Hoang Nguyen, Sy Van Hoang, Khoi Minh Le, Thu Trang Nguyen, Vuong Lam Nguyen, Bac Hoang Nguyen, Binh Quang Truong

**Affiliations:** 1 Department of Cardiovascular Surgery, University Medical Center, University of Medicine and Pharmacy at Ho Chi Minh City, Ho Chi Minh City, Vietnam; 2 Ho Chi Minh City, Vietnam; 3 Department of Internal Medicine, University of Medicine and Pharmacy at Ho Chi Minh City, Ho Chi Minh City, Vietnam; 4 Department of Surgery, University of Medicine and Pharmacy at Ho Chi Minh City, Ho Chi Minh City, Vietnam

**Keywords:** Learning curve, Minimally invasive cardiac surgery, Mitral valve surgery

## Abstract

**Background:**

Minimally invasive mitral valve surgery is becoming a gold standard and provides many advantages for patients. A learning curve is required for a surgeon to become proficient, and the exact number to overcome this curve is controversial. Our study aimed to define this number for mitral valve surgery in general, for replacement and repair separately.

**Methods:**

A total of 204 mitral valve surgeries were performed via the right minithoracotomy approach from October 2014 to January 2019 by a single surgeon who isexperienced in conventional mitral valve surgery. Learning curves were analysed based on the trend of important variables (cross-clamp time, CPB time, ventilation time, ICU time, composite technical failure) over time, and the number of operations required was calculated by CUSUM method.

**Results:**

MIMVS provided an excellent outcome in the carefully selected patients, with low mortality of 0.5% and low rate of complications. The decreasing trend of the important variables were observed over the years and as the cumulative number of procedures increased. The number of operations required to overcome the learning curve was 75 to 100 cases. When considered separately, the quantity for mitral valve replacement was 60 cases, whereas valve repair necessitated at least 90 cases to have an acceptable technical complication rate.

**Conclusion:**

MIMVS is an excellent choice for mitral valve surgery. However, this approach required a long learning curve for a surgeon who is experienced in conventional mitral valve surgery.

**Trial registration:**

The research was registered and approved by the ethical board of the University of Medicine and Pharmacy at Ho Chi Minh City, number 141/DHYD-HDDD, on April 11th 2018.

## Introduction

Minimally invasive mitral valve surgery has been adopted for nearly 3 decades. Since the first successful case performed by Carpentier [1], this approach has witnessed a gradual and steady increase in quantity as well as quality. It has become a routine approach for mitral valve procedures in many centers. The advantages of minimally invasive approach via the right minithoracotomy for both mitral repair and replacement have been proved by many authors [2, 3]: Less bleeding, less transfusions, no sternal wound infection, reducing the time to return to normal life and improve cosmesis without compromising the short term and long term outcome [4].

However, there are also disadvantages regarding this novel approach, including prolonged cardiopulmonary bypass time, prolonged cross-clamp time and an increase in stroke and aortic dissection rate [4]. To achieve a good result, cardiac surgeons need to overcome a substantial learning curve of 75 to 125 operations [5]. This large number creates a reluctancy to apply the new technique for mitral valve surgery. Nevertheless, there are still controversies in the number of cases required to have a good result and the authors did not separate mitral repair and mitral replacement [6, 7].

Therefore, the purpose of this study is to assess the common learning curve as well as the specific curves for mitral valve repair and mitral valve replacement and supply additional information for this interesting topic.

## Material and methods

### Patient selection

From October 2014 to January 2019, a total number of 204 patients underwent minimally invasive mitral valve surgery (MIMVS) at our institute met the inclusion criteria. The operations are performed by a single surgeon who were specialized in adult cardiac surgery before starting the minimally invasive program. We excluded patients with more than moderate aortic regurgitation, concomitant aortic valve disease requiring operation, a history of right chest operation or chest irradiation, severe aortoiliac stenotic diseases and prior cardiac surgery. Preoperative, operative and postoperative data was collected prospectively and analysed.

The research was approved by the ethical board of the University of Medicine and Pharmacy at Ho Chi Minh City, number 141/DHYD-HDDD, on April 11th 2018.

### Surgical technique

All operations are performed by a single surgeon who is experienced in mitral valve surgery via conventional sternotomy.

The patient was placed on the supine position with a cushion under the right scapula to facilitate exposing the two atria. Single lumen endotracheal tube intubation was used. Cardiopulmonary bypass was set up with cannulation of the femoral artery and vein through a small incision in the right groin. A 4–5 cm skin incision was made parallel to the anterior axillary line. A video camera was inserted through a 5 mm port in the third right intercostal space. The transthoracic Chitwood aortic cross-clamp was inserted and aortic clamping was performed. Two liters of Custodiol HTK solution was delivered antegradely into the aortic root through a long metal cardioplegic needle and repeated every 120 min if necessary. A left atriotomy is performed and a sump suction is inserted to keep the surgical field cleared. A left atrial retractor was used to expose the mitral valve. We then assessed the mitral valve for the feasibility of repairing, with the minimally invasive approach, nearly all mitral plasty techniques could be applied, in our center, we regularly used the plication technique, artificial chordae, loop technique [8], a rigid annuloplasty ring was implanted to support the repair. If the valve was severely damaged and repair was not achievable, mitral valve replacement would be performed. Transesophageal Echocardiography (TEE) was used to control the result of the operation.

**Fig. 1:**
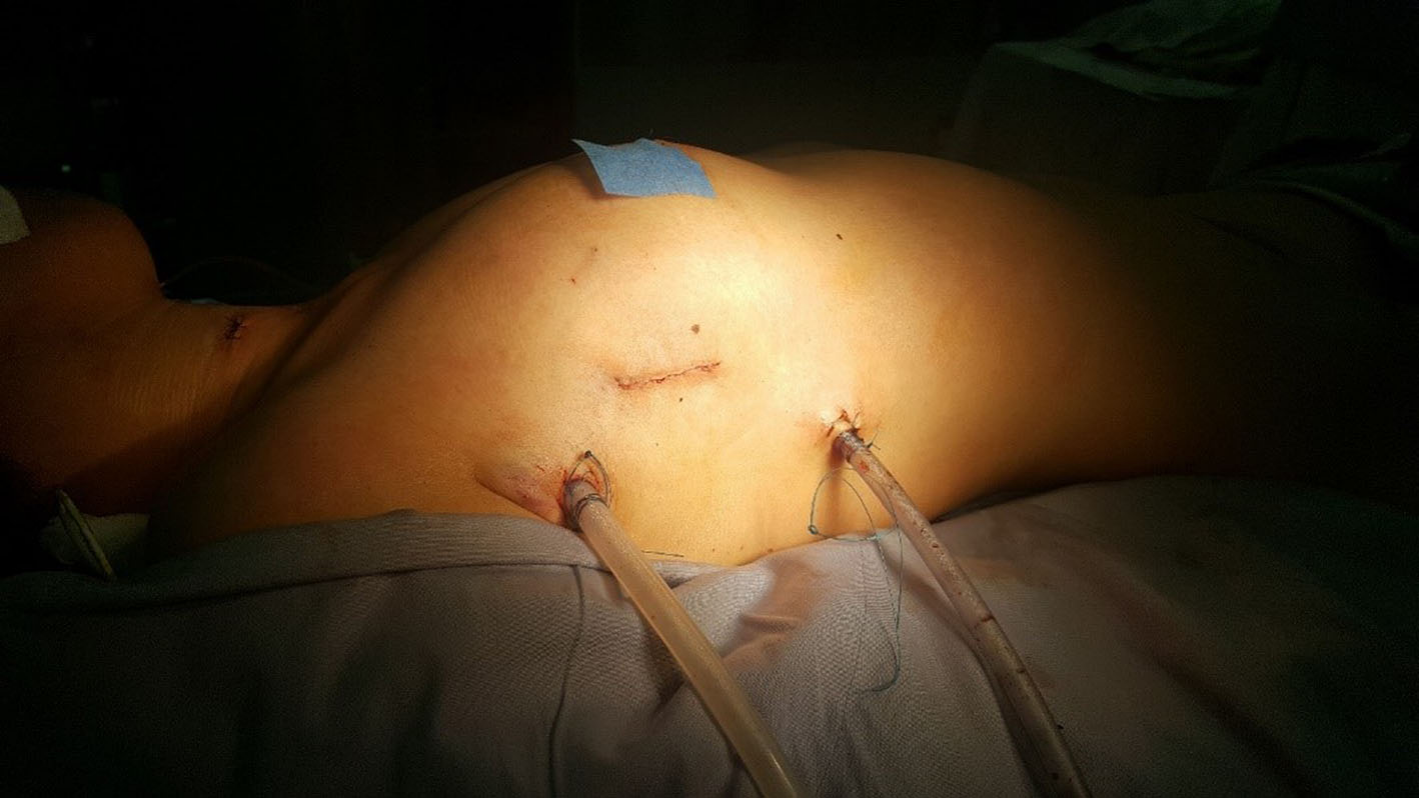
The skin incision after surgery

In case of tricuspid valve repair, the annuloplasty with a tricuspid rigid ring (Carpentier – Edwards ring, Edwards Lifesiences, Irvine, CA, U.S) was used. Concomitant Maze procedure was performed with a long monopolar radiofrequency malleable probe (Fig. 1).

### Definition of technical failure

Based on Wu et al.’s criteria for technical failure [6], we lightly modified the definitions to match our population. Technical failure was defined with the presence of one or more of the events below:

- Perioperative death.
- Conversion to sternotomy.
- More than mild mitral regurgitation on intraoperative TEE in mitral valve repair.
- More than mild paravalvular leak on intraoperative TEE in mitral valve replacement.
- Aortic dissection.
- Reoperation for any reason.
- Wound infection.
- Femoral vessels stenosis or perforation.

### Data analysis

Continuous variables are expressed as mean ± SD and categorical variables are expressed as proportion. Early mortality and complications together with mid-term results, including mortality, recurrent rate and reoperation rate are collected and analysed.

For the learning curve, according to Holzhey et al. [5], we divided patients into groups of 50 successive cases to analyse the difference of the following variables between groups using the χ2 test:

- Cross clamp time.
- Cardiopulmonary time
- Mechanical ventilation time
- Technical failure rate.
- Concomitant procedure rate.

The trend of these variables over the years was also evaluated.

The cumulative sum (CUSUM) failure analysis was used to determine the number of cases needed to overcome the learning curve. This method has been described recently to generate the learning curves for new techniques [6, 9]. We applied the statistical principles from Rogers et al. [10]. CUSUM was defined as Sn = ∑(*Xi* − *p*0), where Xi = 0 for success and Xi = 1 for failure, p0 is the target reference. In this study, p0 was chosen at 0.1, meaning the acceptable composite failure rate was 10%, and a p1 = 0.2 was also as an unacceptable complication rate according to previous publications [5, 6]. Based on p1 and p2, control boundaries were calculated and drawn (false-positive α = 0.05, false-negative β = 0,05). When the curve crosses the upper boundary, the surgeon needs to revise his technique and result. When the curve crossed the lower boundary, the complication rate of the surgeon is equal or below the acceptable rate, meaning the learning curve is achieved. We analysed the CUSUM analysis for the whole group as well as the replacement and repair group separately.

## Results

From October 2014 to January 2019, 204 patients underwent MIMVS, performed by one surgeon, met the inclusion criteria. Baseline patients characteristics are shown in Tables 1 and 2.

**Table 1:** Baseline patients’ characteristics

Variables	Number (*n* = 204)
Age	48.5 ± 24.9
Male, n (%)	97 (47.5)
Post rheumatic, n (%)	91 (44,6)
Degenerative, n (%)	110 (53,9)
Endocarditis, n (%)	3 (1,5)
Preoperative atrial fibrillation, n (%)	51 (25)
Hypertension, n (%)	60 (29.4)
Type 2 diabetes, n (%)	9 (4.4)
Mean left ventricle ejection fraction (LVEF), %	62.8 ± 15.1
Mean EuroSCORE II, %	1.3 ± 0.7
NYHA class	
I, %	6
II, %	80
III, %	12
IV, %	2

**Table 2:** Mitral valve regurgitation distribution according to Carpentier classification

Regurgitation type	N (%), (*n* = 123)
Type I	3 (2.4)
Type II	110 (89.4)
Type IIIA	10 (8.2)
Type IIIB	0 (0)

In 110 patients with type II regurgitation, there were 63% patients with posterior leaflet prolapse, 22% patients with anterior prolapse and 15% patients developed bileaflet prolapse.

Intraoperative variables and postoperative complications are listed in Table 3.

**Table 3:** Intraoperative variables and postoperative outcomes

Intraoperative variables	
Repair rate, n (%)	90 (44)
Repair rate in degenerative disease, n (%)	86 (78.2)
Concomitant procedures	
-Tricuspid valve repair, n (%)	35 (17.2)
-Maze procedure, n (%)	17 (8.3)
-ASD closure, n (%)	2 (1)
Mechanical valve used, n (%)	71 (62)
Mean valve size (mm)	27.1 ± 3.2
Mean cross-clamp time	103 ± 30.2
Mean cardiopulmonary bypass (CPB) time	155.6 ± 42.3
Conversion rate (%)	1.5
Postoperative	
30-day mortality, n (%)	1 (0.5)
Low cardiac output syndrome	
-Intraaortic balloon pump (IABP), n (%)	3 (1.5)
-ECMO, n (%)	0 (0)
Myocardial infarction, n (%)	1 (0.5)
Reoperation for bleeding, n (%)	5 (2.4)
Recoverable stroke, n (%)	1 (0.5)
Renal failure required dialysis, n (%)	0 (0)

One patient with mechanical valve replacement died during the ICU time due to cerebral hemorrhage caused by coagulation disorders Table 4.

**Table 4:** Technical failure events

Variables	*N* = 204
Early mortality, n (%)	1 (0.5)
Conversion to sternotomy	3 (1.5)
Reoperation for bleeding	5 (2.5)
Femoral vessels complications	2 (1)

The mean follow-up time was 34.2 months, during this time ten patients lost follow up Table 5.

**Table 5:** Mid-term results

Variables	*N* = 193
Mortality, n (%)	1 (0.5)
Recurrent mitral disease requiring reoperation, n (%)	2 (1)
Reoperation, n (%)	2 (1)

In the recurrent mitral disease requiring reoperation, one patient had mechanical valve obstruction due tue thrombosis and one patient had a recurrent severe mitral regurgitation.

### Learning curve analysis

**Fig. 2:**
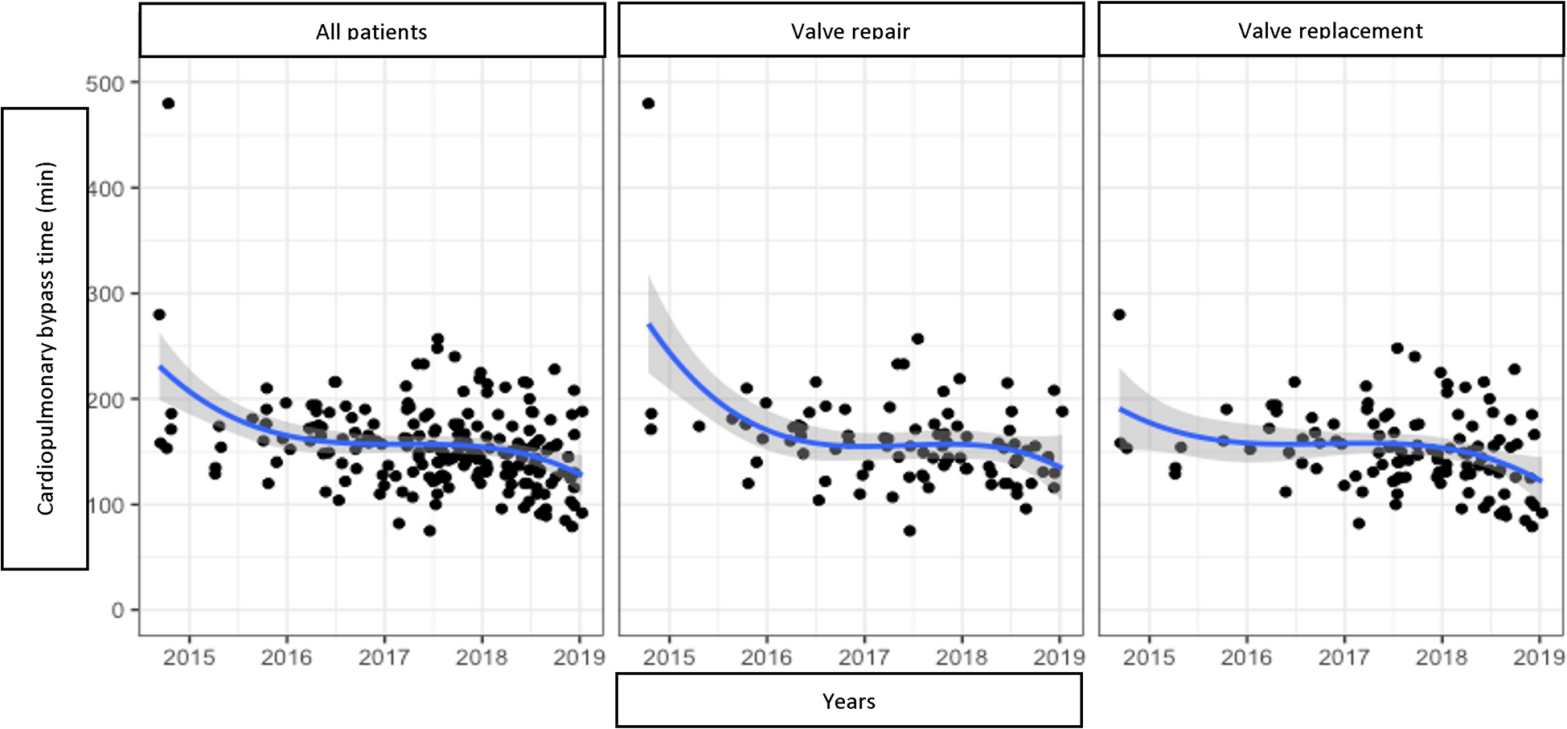
Cadiopulmonary bypass time trend by years

**Fig. 3:**
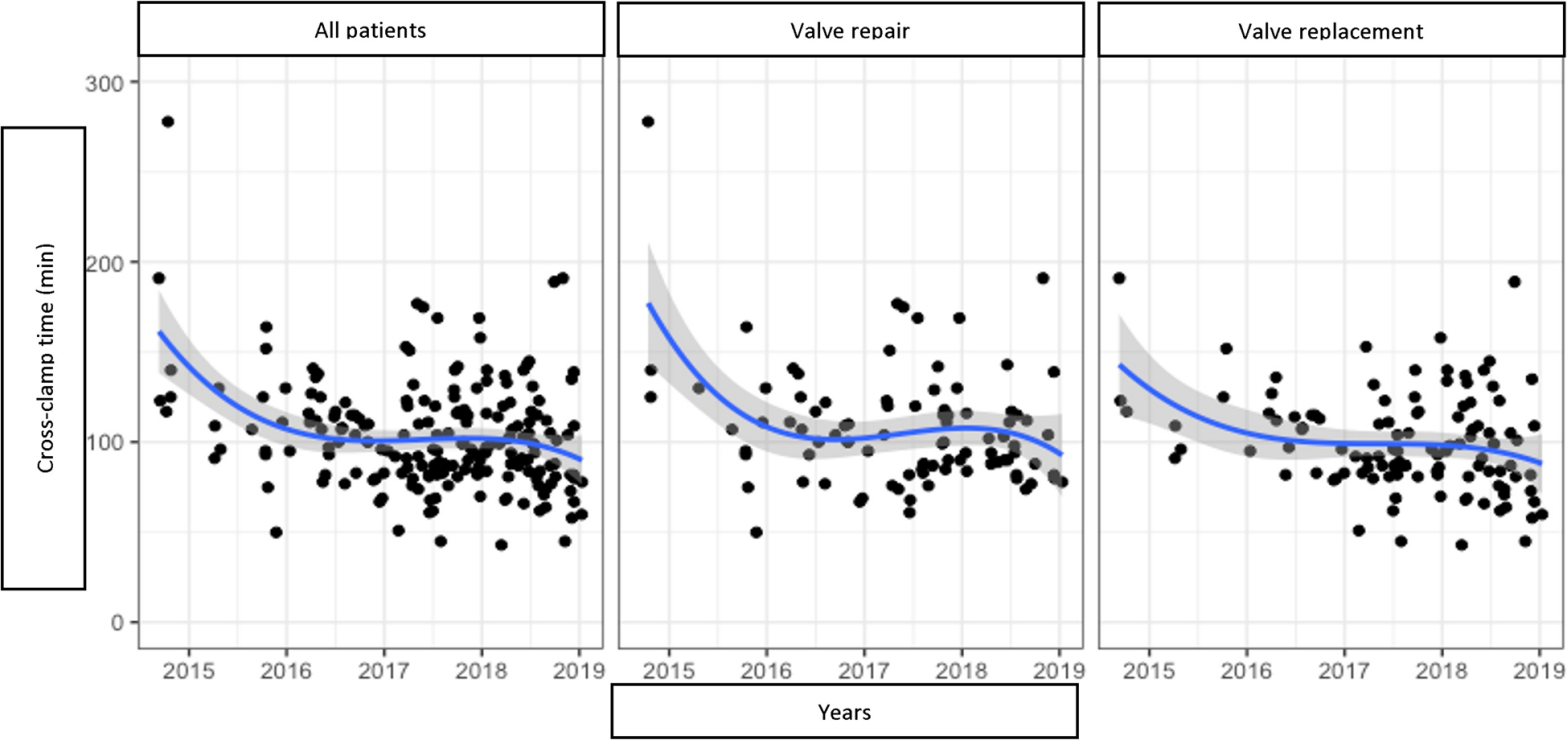
Cross-clamp time trend by years

**Fig. 4:**
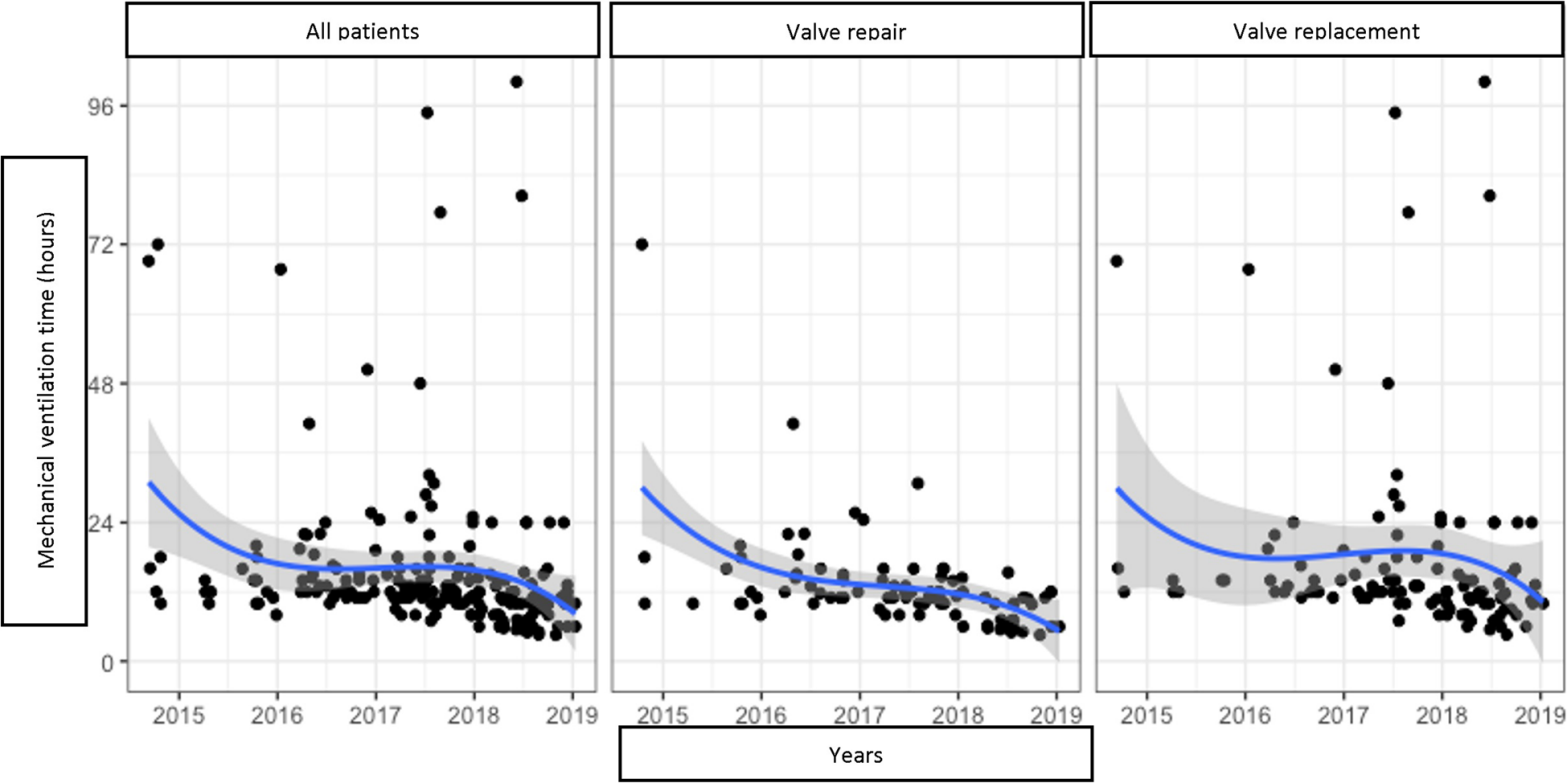
Mechanical ventilation time trend by years

**Fig. 5:**
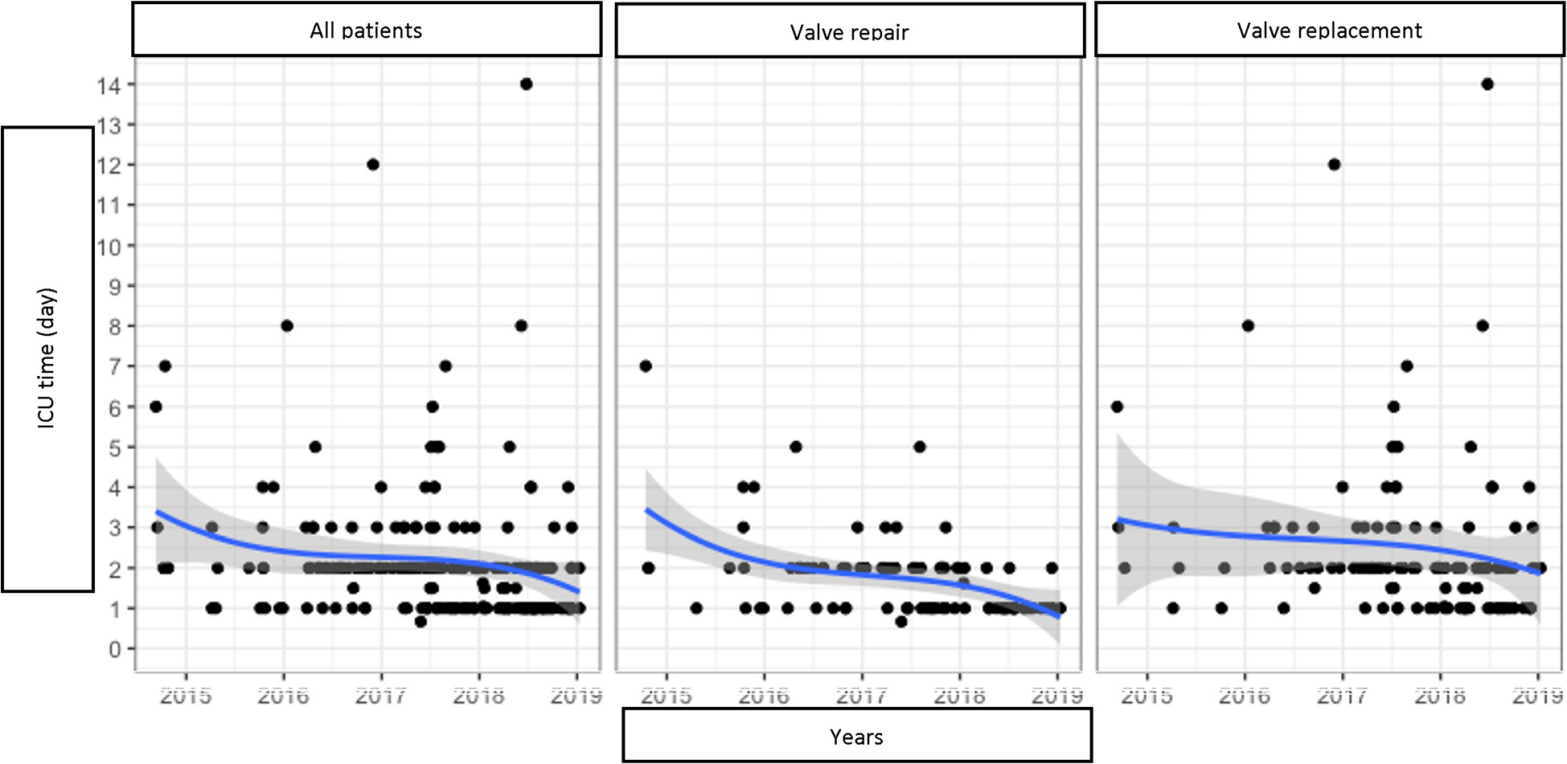
ICU time trend by years

We analysed the trends of the following variables over the years (Figs. 2, 3, 4 and 5):

- CPB time.
- Cross-clamp time.
- Mechanical ventilation time.
- ICU time.

Applying Holzhey’s method [5], we divided patients into groups of 50 consecutive operations to assess the trends of these variations, the concomitant procedure rate and composite technical failure rate Table 6.

**Table 6:** Important variables divided by groups of 50 patients

Variables	1–50	51–100	101–150	151–200	*P*
Cross-clamp time (min)	115,1 (33,3)	104,8 (29,5)	99,2 (24,8)	95,5 (29,6)	0.0003^a^
CPB time (min)	173,9 (53,3)	155,6 (39,5)	151,9 (31,6)	143,3 (36,6)	0.0009^a^
Ventilation time (hours)	18,0 (14,2)	16,8 (10,4)	10.4 (7,2)	8,7 (4,9)	< 0.0001^a^
ICU time (hours)	56,4 (35,7)	61,3 (43,8)	46,8 (23,5)	35,7 (19,2)	0.0001^a^
Concomitant procedures	14,0%	28,0%	30,0%	30,0%	0.0326^b^
Composite technical failure rate	16,0%	20,0%	9,0%	8,0%	0.0492^b^

^a^ Kruskal Wallis test

^b^ Chi square test

**Fig. 6:**
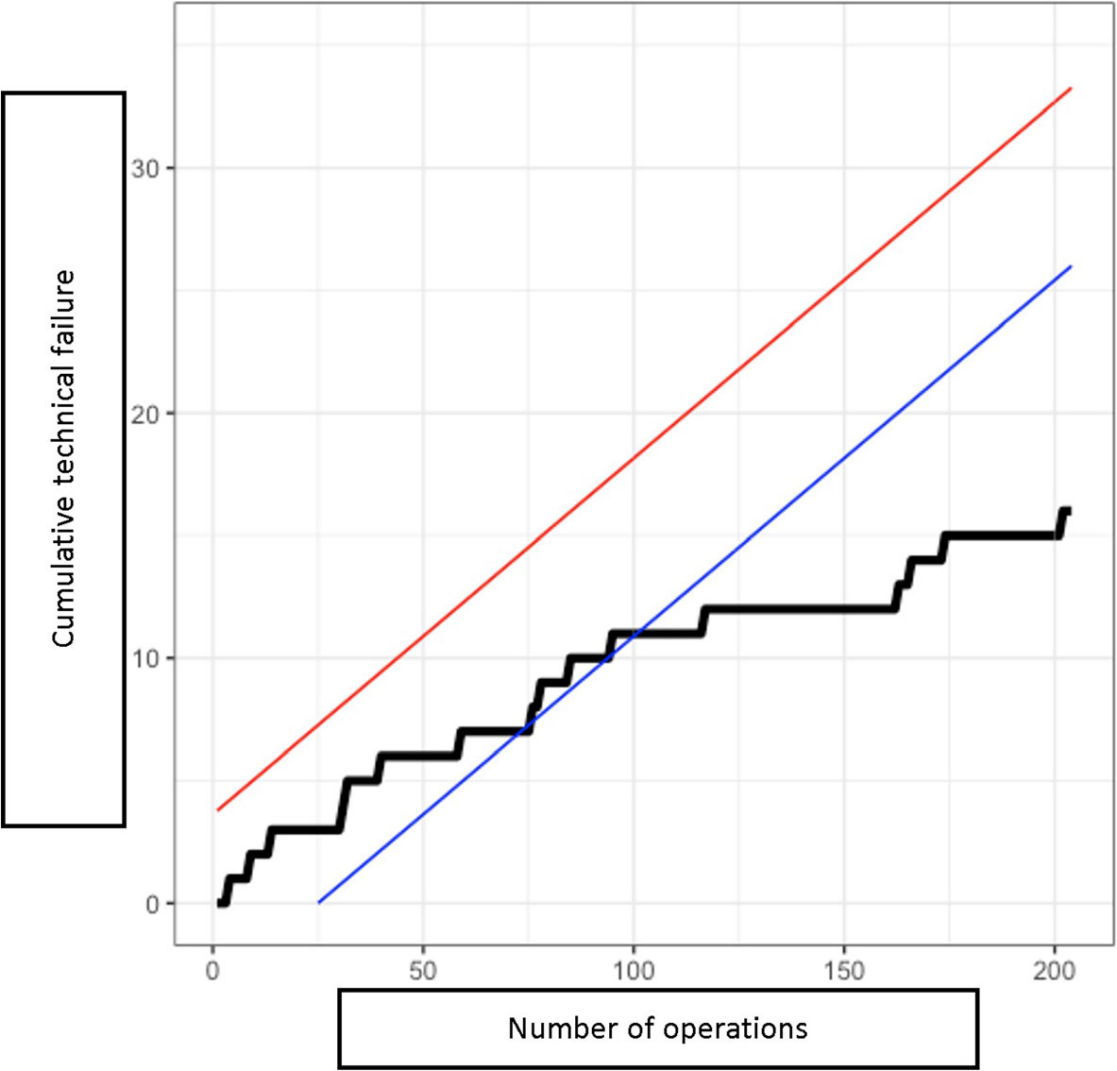
Overall CUSUM learning curve in MIMVS

**Fig. 7:**
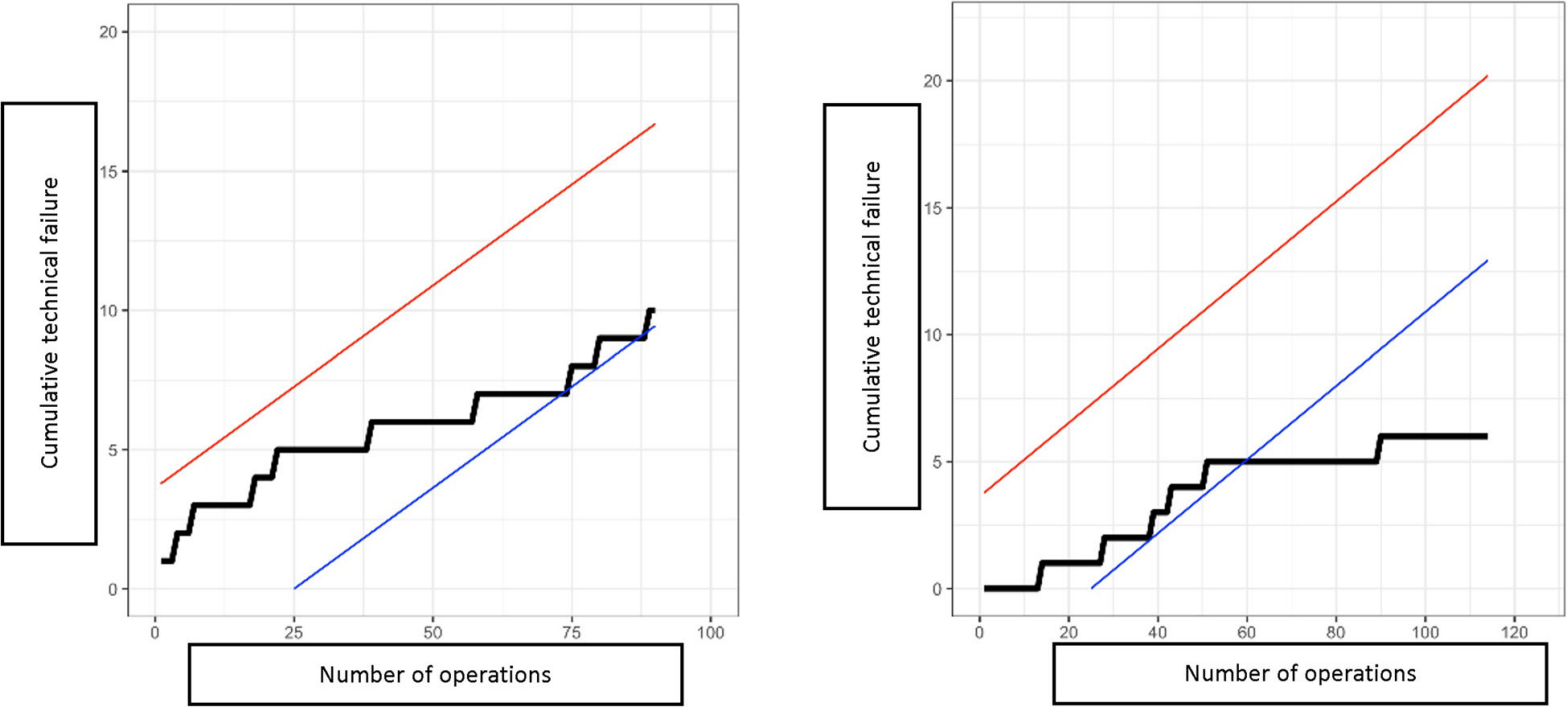
CUSUM learning curve in mitral valve repair (Left) and mitral valve replacement (Right)

This statistical analysis showed a gradual decrease in the important variables toward a positive results trend in MIMVS as the accumulated number of cases increased in time (Figs. 6 and 7).

The overall CUSUM learning curve showed that at the level of 75 cases, the curve started to contact the blue lower boundary and crossed the boundary at the level of 100 cases. However, the learning process seemed to be faster for mitral valve replacement. In this case, CUSUM curve contacted the lower boundary at 40 cases and passed the boundary at 60 cases. On the contrary, the learning curve of mitral valve repair appeared to be longer, it contacted the blue boundary at the 75 cases but fluctuated significantly. Unfortunately, the number of cases were not enough to assess the point where the curve passed the blue line, but at least at 90 cases, the curve had not passed the lower boundary.

## Discussion

Over the past 20 years, the tremendous growth of available transcatheter procedures (i.e. Transcatheter Aortic Valve Replacement - TAVR, Transcatheter mitral valve repair …) and the demand of less invasive approach for cardiac surgery has created a new tendency in this area, the minimally invasive trend. The benefit of the minimally invasive cardiac surgery has been proved by many authors: similar mortality and complication rate when compared with the conventional sternotomy, decreased blood loss and decreased ICU time [3, 4]. Nevertheless, this method also has some disadvantages, including prolonged CPB, cross-clamp time, and a controversial increase in stroke and aortic dissection [4, 11–13].

Our result confirmed the benefits of minimally invasive mitral valve surgery, as shown by previous studies. It showed a low early mortality, low complication rate, short mechanical ventilation time, short ICU and postoperative time, with a relatively low conversion rate.

However, when a new technique is developed, a learning curve is needed for a surgeon to gain experience and confidence. In this research, we have witnessed a significant decrease of CPB and cross-clamp time over the years and as the cumulative number of cases increased. When the surgeon’s experience and confidence developed, the rate of concomitant procedures increased and technical complications decreased remarkably. Nissen et al. also found out that a growth in volume improved the results and shortened CPB and cross-clamp time in both minimally invasive mitral and aortic valve surgery [14]. Holzhey et al. showed a decrease of adverse events from 25% to around 10% after an average of 250 operations, particularly reoperation for bleeding and conversion to sternotomy [5]. However, these results were somehow almost a certainty since everyone could give a conclusion that an average surgeon would perform better case after case.

To give a better concept, many authors had assessed the learning curve in MIMVS to find the average number of cases to overcome it. There are many tools to analyse this curve, including CUSUM method. The CUSUM technique helps tracking changes in early mortality and technical complications. This method has been used by many authors to calculate the required number of cases when a new technique was adapted [5, 6, 10]. Recently, CUSUM technique has been applied in MIMVS and the results were largely varied among authors.

Holzhey et al. showed a large number of 75 to 125 cases to overcome the learning curve and preserve good outcome in MIMVS. The authors also recommended a frequency of at least 2 cases per week to maintain the results [5]. However, this number fluctuated remarkably, Wu et al. showed that the number of cases required was only 33 cases [6]. A survey with 20 experienced surgeons conducted by Misfeld et al. showed an interesting result: To overcome the learning curve, 90% of surgeons believed that 20 cases were sufficient; exceptionally, 2 surgeons thought that 10 cases were good enough [7]. Our number, 75 to 100 cases to overcome the curve, supported Holzhey’s results. This was, however, a fairly large number for many centers, taken into account the low average number of mitral valve surgery per surgeon per year in the United States. We believed this was the necessary quantity of cases to become proficient in MIMVS. With a large amount of mitral valve diseases, Asian countries could make a big step forward in this approach, and large centers could be able to provide an effective and well-established MIMVS fellowship for young colleagues from the other side of the world.

On another point, separating mitral valve repair and mitral valve replacement while analysing the learning curve is neccessary. Mitral valve repair is more demanding in terms of techniques, whereas mitral valve replacement is similar in different patients. Our CUSUM analysis demonstrated the number of cases required to overcome the learning curve in mitral valve replacement are much lower than that of mitral valve repair, 60 cases versus 100 cases, respectively. This proved that it is easier for a surgeon to begin with mitral valve replacement via right minithoracotomy. When the operator becomes more comfortable, simple repairs (i.e. isolated P2 prolapse) could be performed, therefore shortened the learning curve.

One of the questions for MIMVS was the availability of mitral valve repair techniques. Authors proved this approach could attain an equal repair result as conventional sternotomy despite small surgical field [15, 16]. Almost all repair techniques could be applied in MIMVS, with an equal or even better exposure of the valve leaflets and apparatus [2].

Besides the number of operations, patient selection also plays an important role in starting the MIMVS program. We began with patients with isolated mitral valve disease, good left ventricular function, no pulmonary hypertension, no aortic regurgitation and no severe comorbidities, while severe patients were reserved for sternotomy in the initial phase. Nissen et al. also emphasized that carefully patient selection on the basis of many parameters was critical [14].

### Limitations

This study only reported a single surgeon’s experience in one center and the number of cases were still limited. This was not a comparative design, as a result, a definitive conclusion could not be made.

## Conclusions

MIMVS is an excellent choice for mitral valve surgery and provides an excellent short term outcome. However, this approach required a long learning curve of 75 to 100 cases for a surgeon who is experienced in conventional mitral valve surgery. Minimally invasive mitral valve replacement had a lower number of cases required to overcome the curve in comparison with mitral valve repair. Patient selection also played an important role in the initial phase of the program.

## Data Availability

The datasets used and/or analysed during the current study are available from the corresponding author on reasonable request.

## Abbreviations

CPB: Cadiopulmonary bypass
CUSUM: Cumulative sum
ICU: Intensive Care Unit
MIMVS: Minimally invasive mitral valve surgery
TAVR: Transcatheter aortic valve replacement
TEE: Transeophageal Echocardiography
TTE: Transthoracic Echocardiography
